# SARS-CoV-2 promote autophagy to suppress type I interferon response

**DOI:** 10.1038/s41392-021-00574-8

**Published:** 2021-05-08

**Authors:** Xianfeng Hui, Linliang Zhang, Lei Cao, Kun Huang, Ya Zhao, Yufei Zhang, Xi Chen, Xian Lin, Mingzhou Chen, Meilin Jin

**Affiliations:** 1 State Key Laboratory of Agricultural Microbiology, Huazhong Agricultural University, Wuhan, China; 2 State Key Laboratory of Virology and Modern Virology Research Center, College of Life Sciences, Wuhan University, Wuhan, China; 3 CAS Key Laboratory of Special Pathogens and Biosafety, Wuhan Institute of Virology, Wuhan, China

**Keywords:** Microbiology, Cell biology

**Dear Editor**,

The outbreak of SARS-CoV-2 leads global epidemic with high morbidity and mortality. However, the pathophysiology of this deadly virus is complex and largely unknown. Autophagy is a highly conserved homeostatic process that allows cells to recycle their components. Several studies provided evidence that human coronavirus infections are closely related to various cellular aspects associated with autophagy.^1^ Autophagy may play a crucial role in the SARS-CoV-2 viral lifecycle.

In order to investigate whether the autophagy is altered in response to SARS-CoV-2 infection, we infected GFP-LC3 transfected Vero-E6 cells at MOI of 0.05. In comparation to uninfected cells, SARS-CoV-2 infected cells showed a strong increase of GFP-LC3 positive autophagosomes (Fig. 1a). This was also observed in SARS-CoV-2 infected Huh7.0 cells (Supplementary Fig. S1a). The enhanced autophagosome formation was also validated by detecting lapidated LC3-II increased by SARS-CoV-2 infection at 12, 24, and 48 h postinfection (hpi) in Vero-E6 cells (Fig. 1b), Huh7.0 cells (Supplementary Fig. S1b), and Caco-2 cells (Supplementary Fig. S1c). Ultrastructural analysis of SARS-CoV-2 infected Vero-E6 cells by transmission electron microscopy further substantiated the formation of autophagy (Fig. 1c).

**Fig. 1:**
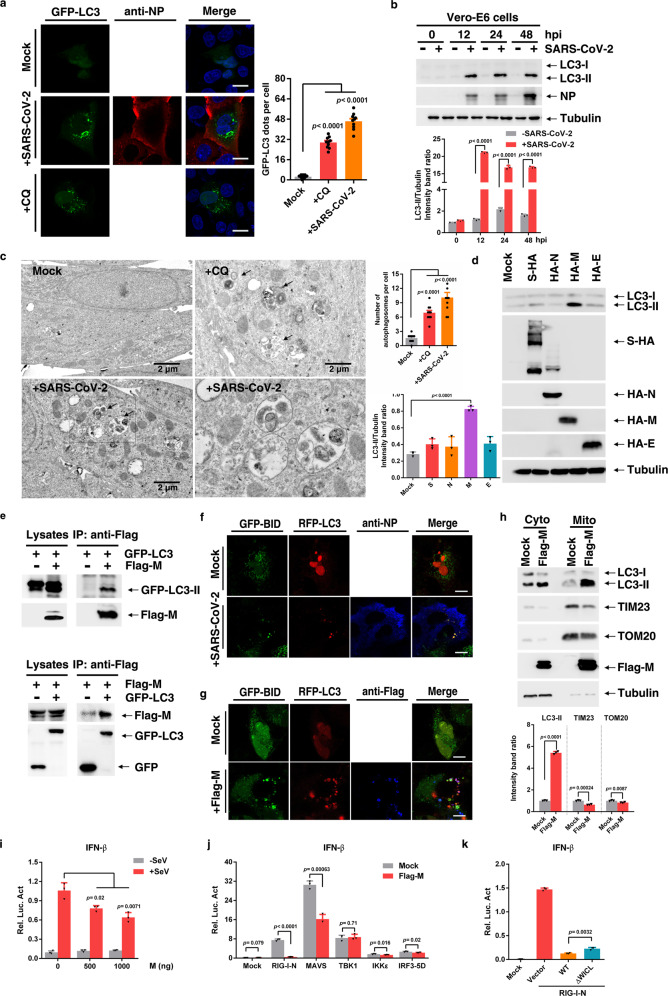
SARS-CoV-2 promote autophagy to suppress type I interferon response. **a** GFP-LC3 dot formation in Vero-E6 cells transiently transfected with GFP-LC3 and either left uninfected (Mock) or infected with SARS-CoV-2 (MOI of 0.05) for 48 h or treated with CQ for 4 h. Scale bar, 10 µm. **b** Vero-E6 were uninfected (−) or infected (+) with SARS-CoV-2. Lysates were evaluated by western blotting (WB). **c** EM analysis of Vero-E6 cells that were stimulated with CQ for 4 h, or infected with SARS-CoV-2 (MOI of 0.05) for 24 h. Scale bar, 2 µm. **d** Huh7.0 cells were transfected with vector or S-HA, HA-M, HA-N, or HA-E plasmid. Lysates were analysed by immunoblotting. **e** Interaction between Flag-M and GFP-LC3 in HEK293T cells. **f** Huh7.0 cells were transfected with the indicated plasmids and infected with SARS-CoV-2 and analysed for the co-localization of BID-GFP and RFP-LC3. **g** Huh7.0 cells were transfected with the indicated plasmids and analysed for the co-localization of BID-GFP and RFP-LC3. **h** Huh7.0 cells were transfected with Flag-M plasmid, and mitochondrial fractions were isolated via ultracentrifugation. Cytoplasm (Cyto) and mitochondria (Mito) were analysed by immunoblotting. **i** HEK293T cells were transfected with the indicated plasmids and infected with Sendai virus for 8 h before the reporter assay was conducted. **j** HEK293T cells were transfected with the indicated plasmids, and a reporter assay was conducted after transfection. **k** HEK293T cells were transfected with the indicated plasmids, and a reporter assay was conducted after transfection. Three independent experiments with three technical repetitions were performed. Data are expressed as mean ± SEM (error bars). Statistical analyses used Student’s *t* test. *P* < 0.05 was considered statistically significant

To determine whether individual SARS-CoV-2 protein can induce autophagy, a series of SARS-CoV-2 nonstructural proteins (NSP5, NSP7, NSP8, NSP9, NSP10, NSP12, and NSP13), and main structural proteins (E, M, S, and N) were investigated. The data showed individual nonstructural protein was not able to induce autophagy (Fig S2a). However, M protein could greatly increase the LC3-II formation in Huh7.0 cells (Fig. 1d), and this increase was in a dose-dependent manner (Fig. S2b). Besides, significant increase of GFP-LC3 positive autophagosomes was also found in M-transfected Huh7.0 cells (Supplementary Fig. S2c). Interestingly, when we extended the time after M transfection, we found that M-induced LC3-II peaked at 24 hpt, and then gradually declined, and M seemed to decline along with LC3-II (Fig. S2d). We proposed that the decrease of LC3-II in the late time may be due to the degradation of autophagosome. To validate this, the cells were transfected with M for 24 h and then were treated with CQ for another 12 h to block the autophagosome degradation by lysosome. We found that the LC3-II in CQ-treated cells was strongly increased compared with control (Fig. S2e). It is worth noting that the content of M seems to be closely related to that of LC3-II. Therefore, we assumed that M may be an interaction partner of LC3. The co-immunoprecipitation assay showed that Flag-tagged M can immunoprecipitate GFP-LC3 (Fig. 1e); vice versa, GFP-LC3 can immunoprecipitate Flag-M (Fig. 1e). Endogenous LC3 was also immunoprecipitated by Flag-M in HEK293T cells (Fig. S2f). This collective data show that M is a binding partner of LC3. Usually, binding partners of LC3 family members typically contain an LC3-interacting region (LIR).^2^ LIRs form intermolecular β sheets with LC3 family members by virtue of a consensus W/Fxxl/L motif. We found that a classical WxxL motif exists in M that is most closely corresponds to those found in p62 and ATG13 (Supplementary Fig. S2g). We then made several mutants in LIR motif to determine their interactions with LC3, and found that the interaction of LC3 with M-W31A, M-L34A, M-W31A/L34A, and M with the LIR deletion (M-△LIR) greatly decreased (Supplementary Fig. S2h). However, M-△LIR was still able to promote the autophagy formation, but this effect was attenuated compared with WT-M overexpression (Supplementary Fig. S2i, j). This indicates the LIR motif in M is critical for the interaction with LC3, and the LC3-II formation, but is not necessary for inducing autophagy.

Autophagy can remove organelles upon recognition of autophagic receptors. Therefore, we sought to classify which type of selective autophagy SARS-CoV-2 induced. Transiently expressed M leaded to a significantly decrease of TOM20, TIM23, and p62 in a time dependent manner, but not PA28 (proteasome), L7a (ribosome), and Calnexin (endoplasmic reticulum) (Supplementary Fig. S3a). Besides, we find that M did not alter mitochondrial ROS and dissipate the mitochondrial membrane potential (Supplementary Fig. S6a, b), both of which are closed associated with autophagy. The decrease of the two mitochondrial marker proteins TOM20 and TIM23 were validated after SARS-CoV-2 infection (Supplementary Fig. S3b, c), indicating the turnover of mitochondria. Further result showed that RFP-LC3 was colocalized well with GFP-BID, a mitochondrial marker protein, in SARS-CoV-2 infected cells (Fig. 1f), which suggested mitochondria may be targeted by autophagosome. Because M can induce autophagy, we then determined if M could promote mitophagy. We found that in M-overexpressed Huh7.0 cells, RFP-LC3 was colocalize with GFP-BID. Importantly, we found that M partly colocalized with GFP-BID (Fig. 1g). Besides, our results clearly showed the co-localization of M and mitochondria (mitotracker probes and TOM20 antibody to mark mitochondria) (Supplementary Fig. S3d, e), implying that M may translocate to mitochondria. This was further demonstrated by mitochondrial components separation (Fig. 1h). Of note, in M-expression cells, the level of LC3 in mitochondrial components was significantly higher than control cells, while TOM20 and TIM23 decreased. M-△LIR was also enriched in mitochondrial components, but TOM20 and TIM23 were not significantly changed compared with WT-M (Supplementary Fig. S3f).

Mitochondria is a critical platform to converge antiviral innate immune signaling. Therefore, we attempted to determine whether M protein can disrupt type I interferon response. We found M overexpression significantly inhibited SeV-mediated IFN-β activation in a dose-dependent manner (Fig. 1i). Further, we determined that M blocked RIG-I and MAVS, but not TBK1, IKKε, and IRF3-5D-triggered IFN-β promoter activation (Fig. 1j). It indicated that the inhibitory effect of M on IFN-β activation is mainly through MAVS. As expected, M-△LIR, exhibited an slightly weaker inhibitory effect on IFN-β promoter activation, compared with M-WT (Fig. 1k). These results suggest that M can inhibit RIG-MAVS-triggered IFN-β signaling, which is closely associated with its mitophagy induction.

Interestingly, M seems to serve as a receptor to recruit LC3 to mitochondria. Although the TMHMM Server shows that three transmembrane motifs exist in the SARS-CoV-2 M protein, we are more inclined to believe that M can be recruited to mitochondria by one mitochondrial outer membrane protein. In our attempt to identify this protein, we focused on TUFM, which was shown to mediate influenza virus PB1-F2 and human Parainfluenza virus type 3 M protein to induce mitophagy. We found that SARS-CoV-2 M interacts with TUFM (Supplementary Fig. S4a–c), thus suggested that TUFM may critical role in M translocating and inducing mitophagy, which needs further studies.

Here, we reported that SARS-CoV-2 infection could induce autophagy. Actually, blocking autophagy with inhibitors (3-MA and Wortmannin) inhibited viral replication (Supplementary Fig. S5a–d). This result is consistent with that of a previous report showing that CQ, a drug that hinders autophagy completion, effectively inhibits SARS-CoV-2.^3^ The impact of drugs targeting autophagy represents an emerging topic, worth to be considered as a new therapeutic strategy in the context of COVID-19.^4^

Although several studies reports explored the mechanism of virus-triggered autophagy from the initiation to the last step of autophagic process.^5^ But till now, there is no finding regarding how SARS-CoV-2 utilizes autophagy to escape host immune defense. In this study, we reported SARS-CoV-2 M protein induced mitophagy to break the mitochondria networks to block the downstream innate immunity signaling for inhibiting the type I IFN response. This novel finding provided new avenues for the development of therapeutic strategies to combat viral infections and COVID-19.

## Supplementary information

SUPPLEMENTAL MATERIAL
