# Supplementary material for: SARS-CoV-2 promote autophagy to suppress type I interferon response
Source: Signal Transduct Target Ther. 2021 May 8;6:180. doi: 10.1038/s41392-021-00574-8 (PMC8105701; doi:10.1038/s41392-021-00574-8)
Supplement: Supplementary file 1 — SUPPLEMENTAL MATERIAL [file 41392_2021_574_MOESM1_ESM.pdf]

Supplementary Materials for

SARS-CoV-2 promote autophagy to suppress type I interferon response

Xianfeng Hui[1,7], Linliang Zhang[2,7], Lei Cao[1], Kun Huang[1], Ya Zhao[1], Yufei Zhang[1], Xi Chen[1], Xian Lin[3,#], Mingzhou Chen[2,#], and Meilin Jin[1, #]

[1]*State Key Laboratory of Agricultural Microbiology, Huazhong Agricultural University, Wuhan, China*

[2]*State Key Laboratory of Virology and Modern Virology Research Center, College of Life Sciences, Wuhan University, Wuhan, China*

[3]*CAS Key Laboratory of Special Pathogens and Biosafety, Wuhan Institute of Virology, Wuhan, China*

[7]These authors contributed equally: Xianfeng Hui, Linliang Zhang

[#]Correspondence to: jinmeilin@mail.hzau.edu.cn (MJ); chenmz@whu.edu.cn (MC); linxian@wh.iov.cn (XL)

This PDF file includes:

Materials and Methods
Supplementary Figures and Figure legends

Materials and Methods

Cells and Viruses

Human hepatoma cell line (Huh7.0 cells), Vero-E6 cells, Vero cells, the human epithelial cell line (Caco-2 cells), and human cervical cancer cell line (HeLa cells) were maintained in Dulbecco's modified Eagle's medium (DMEM; HyClone). HEK293T cells were maintained in Roswell Park Memorial Institute medium (RPMI; HyClone). Medium was supplemented with 10% foetal bovine serum (FBS; PAN Biotech) at 37°C in a 5% CO_2 humidified atmosphere. SARS-CoV-2 (strain HB-01) was kindly provided by the National Virus Resource Center, Wuhan Institute of Virology, Chinese Academy of Sciences. The complete genome for this SARS-CoV-2 strain was submitted to GISAID (BetaCoV/Wuhan/IVDC-HB-01/2020|EPI-ISL-402119).

Antibodies and Reagents

Rabbit anti-LC3B (cat. no. 3868S) polyclonal antibodies (Abs), anti-Ribosomal protein L7a (cat. no. 2415S), anti-PA28α (cat. no. 2408S) polyclonal antibodies (Abs) were purchased from Cell Signalling Technology. Rabbit anti-p62 (cat. no. PM045) polyclonal Abs and mouse anti-tubulin (cat. no. M175-3) monoclonal Abs were purchased from MBL. Rabbit anti-Calnexin (cat. no. C4731) polyclonal Abs and anti-TUFM polyclonal Abs were purchased from Sigma-Aldrich. Mouse anti-SARS-CoV-2 nucleocapsid antibody was purchased from Sino Biological (cat. no. 40143-MM05). Rabbit anti-TOM20 (cat. no. 11802-1-AP), anti-TIM23 (cat. no. 11123-1-AP) polyclonal Abs and mouse anti-GFP tag (cat. no. 66002-1-Ig) monoclonal Abs were purchased from Proteintech. Mouse anti-Flag tag (cat. no. AE005) and anti-HA tag (cat.

no. AE008) monoclonal Abs were purchased from ABclonal Technology. Horseradish peroxidase (HRP)-conjugated anti-mouse (cat. no. AS003) and anti-rabbit (cat. no. AS014) secondary Abs were purchased from ABclonal Technology. CoraLite 594-conjugated goat anti-mouse (cat. no. SA00013-3) secondary Abs were purchased from Proteintech.

Chloroquine (CQ) (cat. no. HY-17589A), 3-methyladenine (3-MA) (cat. no. HY-19312), Wortmannin (cat. no. HY-10197) and MG132 (cat. no. HY-13259) were purchased from MedChemExpress. Lipofectamine 2000 (cat. no. 11668) were purchased from Invitrogen.

SDS-PAGE and Western Blotting

For the SDS-PAGE assay, protein samples were prepared by using cell lysis buffer for Western blotting and Immunoprecipitation (IP) (Beyotime) that contained an EDTA-free protease inhibitor cocktail (Bimake). After SDS-PAGE separation, proteins were transferred to nitrocellulose membranes (GE Healthcare). After blocking with 2% bovine serum albumin (BSA, Biofrox) in Phosphate Buffered Saline (PBS), the membranes were incubated with corresponding primary antibodies, followed by horseradish peroxidase-conjugated secondary antibodies.

Immunofluorescence Analysis

Huh7.0/Vero-E6 cells were transfected with the indicated plasmids with Lipofectamine 2000 (Invitrogen) according to the manufacturer’s protocol. After transfection for 24 hours, Huh7.0 cells were infected with SARS-CoV-2 at a MOI of 1, and Vero-E6 cells were infected with SARS-CoV-2 at a MOI of 0.05 for 24 or 48 hours. For immunofluorescence analysis, the cells

to be tested were fixed with 4% paraformaldehyde (PFA) at room temperature for 30 min and treated with 0.1% (vol/vol) Triton X-100 for 10 min. The cells were incubated in 2% (vol/vol) BSA for 1 hour at room temperature. Then, the cells were incubated with the designated primary antibody for 2 hours and then with the appropriate Alexa Fluor-conjugated secondary antibody for 1 hour. Finally, DAPI staining was used to visualize DNA according to the purpose of the experiment. The cells were then observed with a ZEISS confocal microscope under a 60 × oil objective.

In Vivo Coimmunoprecipitation

Huh7.0 cells were transfected with the appropriate plasmids with Lipofectamine 2000 according to the manufacturer's protocol, and 36 hours later, cells were harvested and lysed with lysis buffer (50 mM Tris-HCl [pH 7.4], 150 mM NaCl, 1% [wt/vol] Triton X-100, 1 mM EDTA [pH 8.0], 0.1% [vol/vol] sodium dodecyl sulfate [SDS], and protease inhibitor cocktail) for 30 min at 4°C. The supernatants were collected by centrifugation at 13,000 rpm for 30 min at 4°C and precleared by incubation with protein G Sepharose 4 Fast Flow beads for 1 hour at 4°C with rotation. The supernatants were collected via centrifugation at 3,000 rpm for 2 min at 4°C, and specific primary antibodies were added and incubated overnight at 4°C with rotation. The beads were collected via centrifugation at 3,000 rpm for 2 min at 4°C and washed three times with 1 mL lysis buffer. Then, the collected beads were boiled at 100°C for 5 min in 50 µL 2 × SDS protein-loading buffer and analysed via Western blot.

Transmission Electron Microscopy

Vero-E6 cells were infected with SARS-CoV-2 for 24 hours at a MOI of 0.05 or treated with CQ (50 mM) for 6 hours. Then, the cells were fixed with 2.5% glutaraldehyde for 2 hours at room temperature. The cells were harvested and fixed with 2.5% glutaraldehyde on ice for 2 hours followed by fixation in 2% osmium tetroxide, and then, the cells were dehydrated with sequential washes in 50%, 70%, 90%, 95%, and 100% ethanol. Areas containing cells were block-mounted and thinly sliced.

Autophagy Analysis

For analyses involving CQ (50 mM), Huh7.0/Vero-E6 cells were treated for 6 hours before further experiments. For analyses involving 3-MA (5 mM) and Wortmannin (10 μM), Huh7.0 cells were treated for 4 hours and treated again after absorption of SARS-CoV-2 until the samples were harvested. For analyses involving only 3-MA (1 mM), Vero-E6 cells were treated for 4 hours and treated again after absorption of SARS-CoV-2 until the samples were harvested.

Virus Plaque Assay

Huh7.0 cells or Caco-2 cells pretreated with 3-MA, Wortmannin were infected by SARS-CoV-2 for 1 hour at a MOI of 1, and then, the medium was replaced with the same medium as that was used for the pretreatment. Additionally, Vero-E6 cells pretreated with 3-MA or Wortmannin were infected with SARS-CoV-2 for 1 hour at a MOI of 0.05, and then, the medium was replaced with the same medium as that used for the pretreatment. The supernatants were obtained at 24 hours after infection for the virus plaque assay. Briefly, the sample to be tested was diluted in basal DMEM, and confluent Vero cells in 12-well plates were inoculated with

the samples and incubated at 37°C for 1 hour. The cells were washed twice with PBS (HyClone) to remove unbound virus. Subsequently, the cells were covered with 1 mL of 4% sodium carboxymethyl cellulose (containing 3% FBS) per well. After culturing for 72 hours, when the cells showed lesions or plaques, each well was filled with 10% neutral formaldehyde and fixed for more than 4 hours. Finally, plaques were stained with 0.1% crystal violet and counted.

Subcellular Fractionation

Subcellular fractionation assays were performed as previously described [1]. Briefly, Huh7.0 cells were transfected with the indicated plasmids, and 36 hours later, cells were harvested and resuspended in 300 mL mitochondria isolation buffer (10 mM Tris-HCl [pH 7.4], 2 mM $MgCl_2$, 10 mM KCl, and 250 mM sucrose) and homogenized with a Dounce tissue grinder for 20 min at 4°C. The supernatants were collected after centrifugation at 500 g for 10 min at 4°C and then centrifuged for another 5,000 g for 10 min to yield the cytosolic fraction. Precipitates were resuspended in 1 mL mitochondria extraction buffer and collected after centrifugation at 5,000 g for 10 min at 4°C to yield the mitochondrial fraction. Equivalent amounts of protein from each fraction were analysed by Western blot with the indicated antibodies.

Reporter Assays

HEK293T cells cultured in 12-well plates were transfected with a control plasmid or plasmids expressing RIG-I-N, MAVS, TBK1, IKKε, or IRF3-5D, along with IFN-β luciferase reporter plasmids or plasmids expressing viral proteins. After 24 hours of transfection, cells were harvested, and cell lysates were used to measure luciferase levels using a Dual Luciferase

Reporter Assay System (Promega). In addition, Sendai virus was used as a positive stimulus.

Flow Cytometric Analyses

Mitochondria-associated ROS levels were measured by staining cells with MitoSOX (Invitrogen) at 2.5 μM for 30 min at 37 °C. Mitochondria membrane potential was measured using the kit from Invitrogen and performed according to the manufacturer's instructions. Cells were then washed with PBS solution and re-suspended in cold PBS solution for FACS analysis.

Statistical Analysis

Data are expressed as the means ± standard errors of the means (SEMs). Statistical analysis was performed by Student's t test using GraphPad Prism software (version 5.0). A P value equal to or lower than 0.05 was considered significant.

Abbreviation

SARS-CoV-2: Severe Acute Respiratory Syndrome Coronavirus 2; CQ: chloroquine; 3-MA: 3-methyladenine; ATG: autophagy related; p62: sequetosome 1; LC3: microtubule-associated proteins light chain 3; TIM23: mitochondrial import inner membrane translocase subunit; TOM20: mitochondrial 20 kDa outer membrane protein; PA28: proteasome activator 28 subunit; L7a: 60S ribosomal protein L7a. RFP-LC3: LC3 fused to red fluorescent protein (gene); GFP-LC3: LC3 fused to green fluorescent protein (gene); BID: BH3-interacting domain death agonist; WT: wild type; SeV: sendai virus; IFN: interferon; RIG-I: antiviral innate immune response receptor; MAVS: mitochondrial antiviral-signaling protein; TBK1: serine/threonine-

protein kinase; IKKε: inhibitor-κb kinase ε; IRF3-5D: interferon regulatory factor 3 mutant; TUFM: Tu translation elongation factor mitochondrial; MOI: multiplicity of infection; hpi: hours post-infection; hpt: hours post-transfection.

References

1 Ding, B. *et al.* The Matrix Protein of Human Parainfluenza Virus Type 3 Induces Mitophagy that Suppresses Interferon Responses. *Cell Host Microbe* **21**, 538-547 e534, doi:10.1016/j.chom.2017.03.004 (2017).

Supplementary Figures

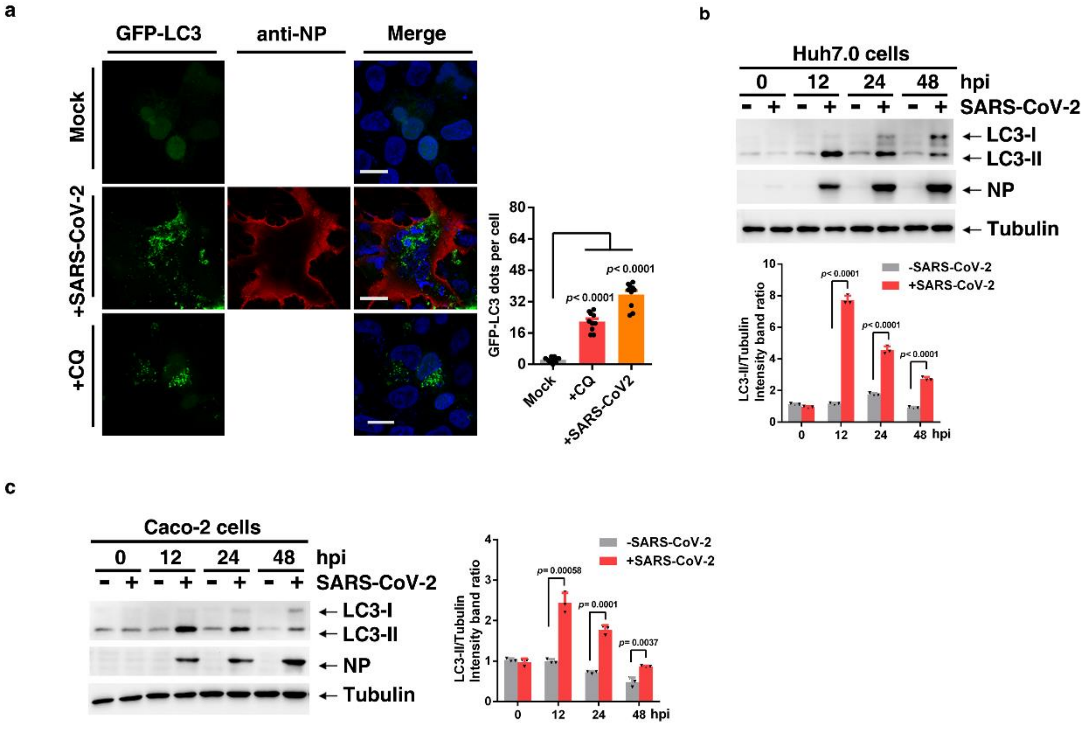

Fig. S1 SARS-CoV-2 infection triggers autophagy in vitro. **a** GFP-LC3 dot formation in Huh7.0 cells transiently transfected with GFP-LC3 and either left uninfected (Mock) or infected with SARS-CoV-2 (MOI of 1) for 48 hours or treated with CQ for 4 hours. Scale bar, 10 µm. **b** Huh7.0 cells were uninfected (-) or infected (+) with SARS-CoV-2. Lysates were evaluated by Western blotting (WB). **c** Caco-2 cells were uninfected (-) or infected (+) with SARS-CoV-2. Lysates were evaluated by Western blotting (WB). Three independent experiments with three technical repetitions were performed. Data are expressed as mean ± SEM (error bars). Statistical analyses used Student's t test. $P < 0.05$ was considered statistically significant.

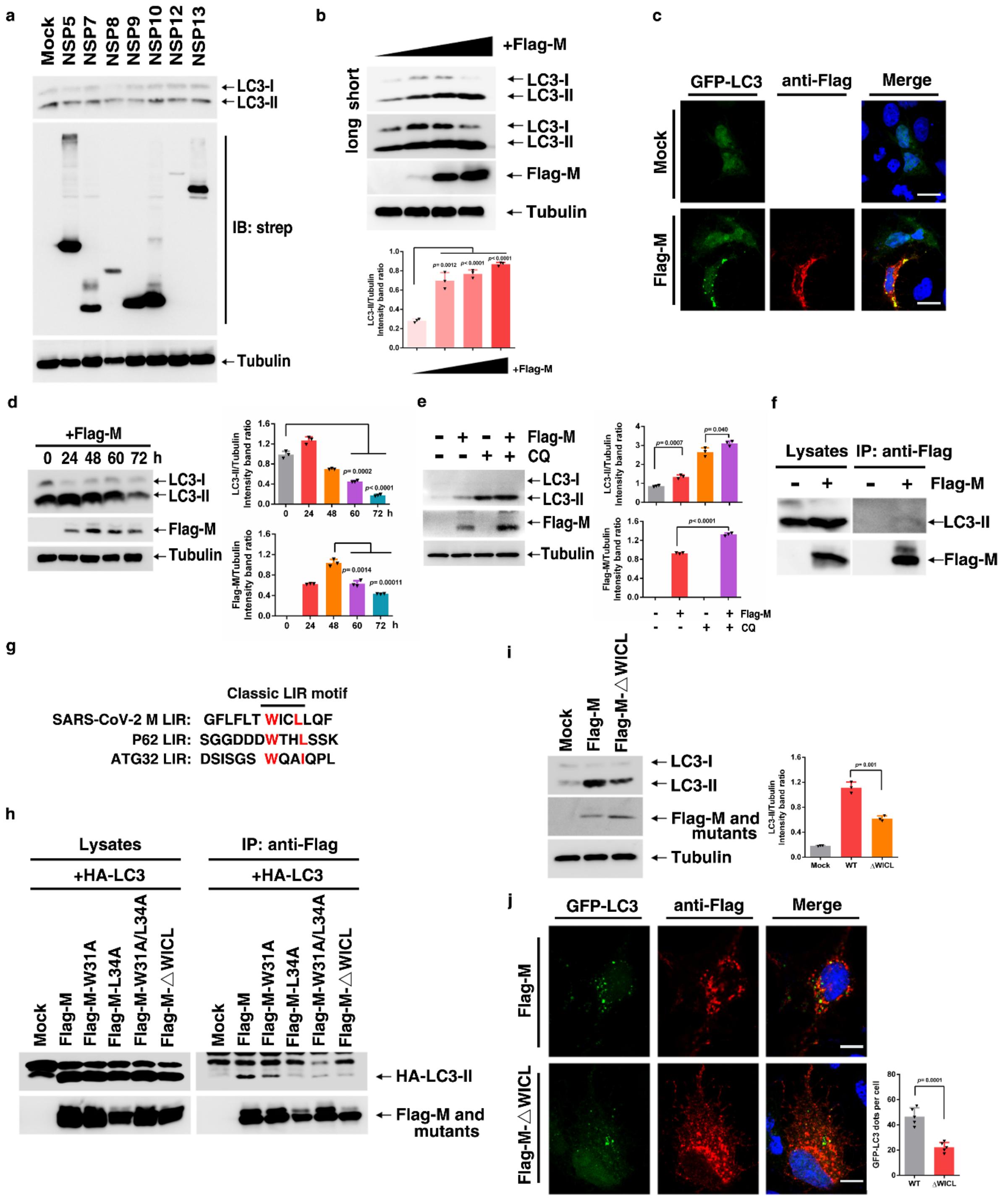

Fig. S2 SARS-CoV-2 M induces autophagy and interact with LC3. **a** Huh7.0 cells were transfected with the indicated plasmids. Lysates were analysed by immunoblotting. **b** Huh7.0 cells were transfected with the indicated plasmids and analysed for the colocalization of Flag-M and GFP-LC3. **c** Huh7.0 cells were transfected with different concentrations of Flag-M plasmid (0 μg, 0.3 μg, 1 μg and 2 μg). Lysates were analysed by immunoblotting. **d** HEK293T

cells were transfected with Flag-M plasmid and collected at different times. Lysates were analysed by immunoblotting. **e** Huh7.0 cells were transfected with Flag-M and treated with CQ. Lysates were analysed by immunoblotting. **f** Interaction between Flag-M and endogenous LC3 in Huh7.0 cells. **g** Typical LIR sequences in SARS-CoV-2-M. **h** Interaction between HA-LC3 and wild-type M and mutants. **i** Huh7.0 cells were transfected with the indicated plasmids and analysed by immunoblotting. **j** Huh7.0 cells were transfected with Flag-M plasmid and mutant plasmids and analysed for GFP-LC3 dots. Scale bar, 10 μm. Three independent experiments with three technical repetitions were performed. Data are expressed as mean ± SEM (error bars). Statistical analyses used Student's t test. $P < 0.05$ was considered statistically significant.

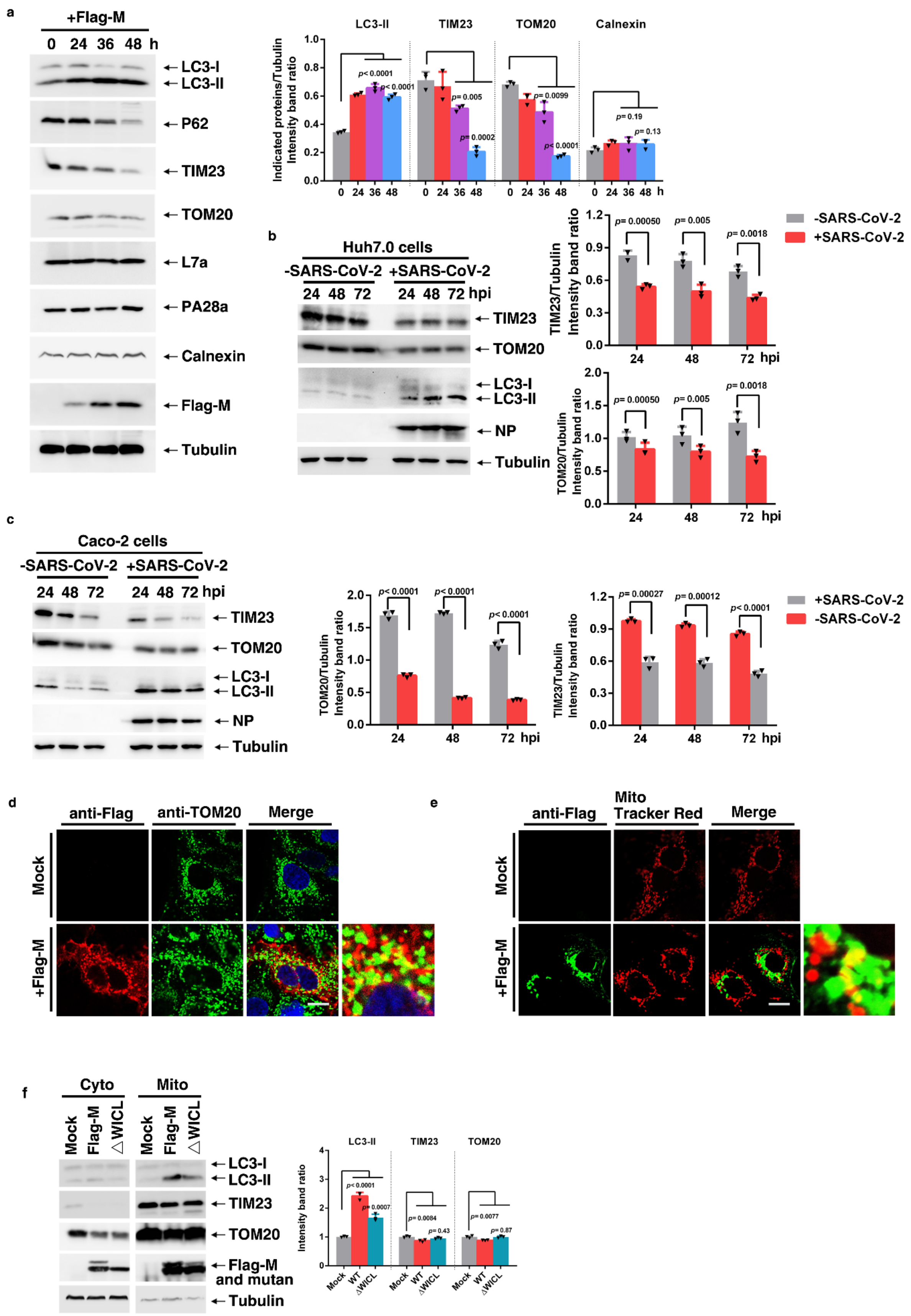

Fig. S3 SARS-CoV-2 and M induces selective mitochondrial autophagy. **a** Huh7.0 cells were

transfected with Flag-M plasmid and collected at different times. Lysates were analysed by immunoblotting. **b** Huh7.0 cells were uninfected (-) or infected (+) SARS-CoV-2. Lysates were collected at different times and analysed by immunoblotting. **c** Caco-2 cells were uninfected (-) or infected (+) SARS-CoV-2. Lysates were collected at different times and analysed by immunoblotting. **d** Huh7.0 cells were transfected with Flag-M plasmid plasmids and analysed for the colocalization of Flag-M and TOM20 (mitochondrial 20 kDa outer membrane protein). Scale bar, 10 μm. **e** Huh7.0 cells were transfected with Flag-M plasmid plasmids and analysed for the colocalization of Flag-M and mitochondria labeled with probes. Scale bar, 10 μm. **f** Huh7.0 cells were transfected with the indicated plasmids, and mitochondrial fractions were isolated via ultracentrifugation. Cytoplasm (Cyto) and mitochondria (Mito) were analysed by immunoblotting. Three independent experiments with three technical repetitions were performed. Data are expressed as mean ± SEM (error bars). Statistical analyses used Student's t test. $P < 0.05$ was considered statistically significant.

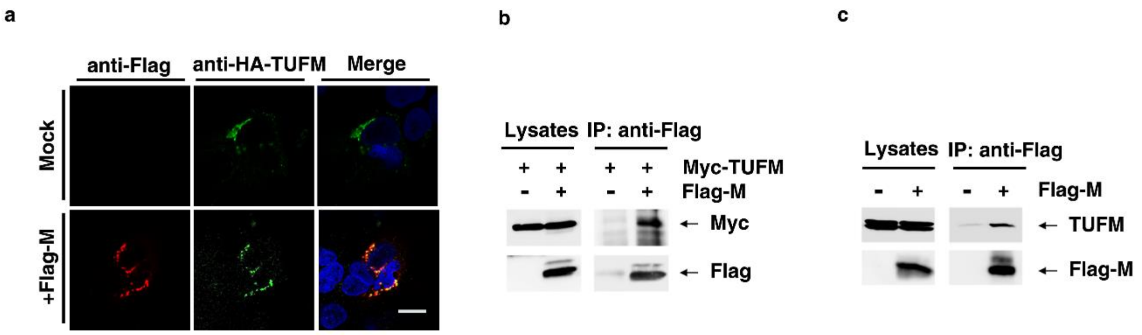

Fig. S4 SARS-CoV-2 M interact with TUFM (Tu translation elongation factor mitochondrial). **a** Huh7.0 cells were transfected with Flag-M plasmid plasmids and HA-TUFM plasmid, analysed for the colocalization of Flag-M and HA-TUFM. Scale bar, 10 μm. **b** Interaction between Flag-M and Myc-TUFM. **c** Interaction between Flag-M and endogenous TUFM in Huh7.0 cells. Three independent experiments with three technical repetitions were performed.

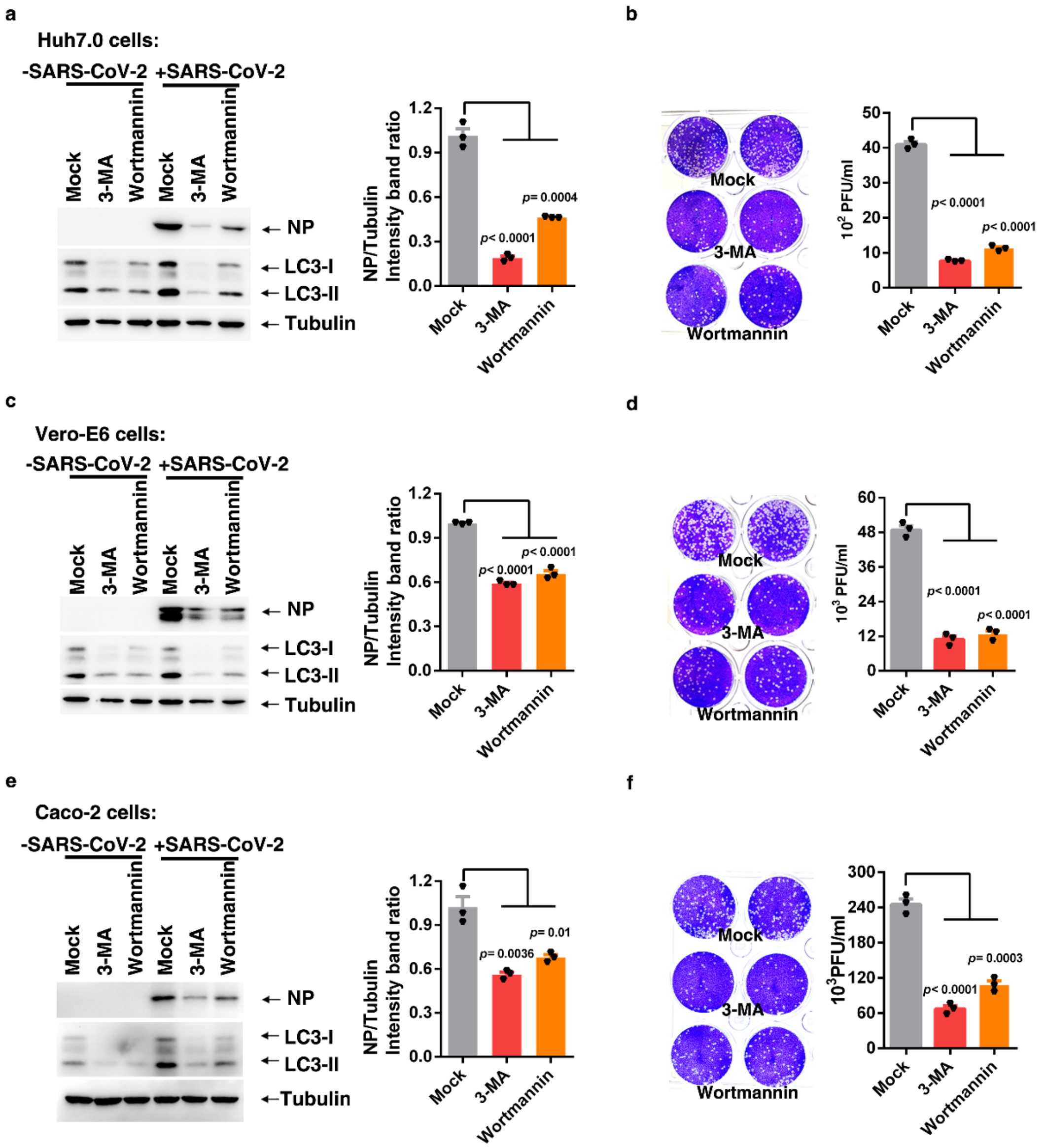

Fig. S5 Blocking autophagy with two pharmaceutical inhibitors (3-MA and Wortmannin) significantly suppresses SARS-CoV-2 infection. **a** Huh7.0 cells were untreated (Mock) or treated with 3-MA/Wortmannin and then uninfected (-) or infected (+) with SARS-CoV-2 (MOI of 1) for 24 hours. Lysates were evaluated by WB. **b** Huh7.0 cells were untreated (Mock) or treated with 3-MA/Wortmannin and then uninfected (-) or infected (+) with SARS-CoV-2 (MOI of 1) for 24 hours. Extracellular viral yields were evaluated by virus plaque assay. **c** Vero-E6 cells were untreated (Mock) or treated with 3-MA/Wortmannin and then uninfected (-) or infected (+) with SARS-CoV-2 (MOI of 0.05) for 24 hours. Lysates were evaluated by WB. **d** Vero-E6 cells were untreated (Mock) or treated with 3-MA/Wortmannin and then uninfected

(-) or infected (+) with SARS-CoV-2 (MOI of 0.05) for 24 hours. Extracellular viral yields were evaluated by virus plaque assay. **e** Caco-2 cells were untreated (Mock) or treated with 3-MA/Wortmannin and then uninfected (-) or infected (+) with SARS-CoV-2 (MOI of 1) for 24 hours. Lysates were evaluated by WB. **f** Caco-2 cells were untreated (Mock) or treated with 3-MA/Wortmannin and then uninfected (-) or infected (+) with SARS-CoV-2 (MOI of 1) for 24 hours. Extracellular viral yields were evaluated by virus plaque assay. Three independent experiments with three technical repetitions were performed. Data are expressed as mean ± SEM (error bars). Statistical analyses used Student's t test. $P < 0.05$ was considered statistically significant.

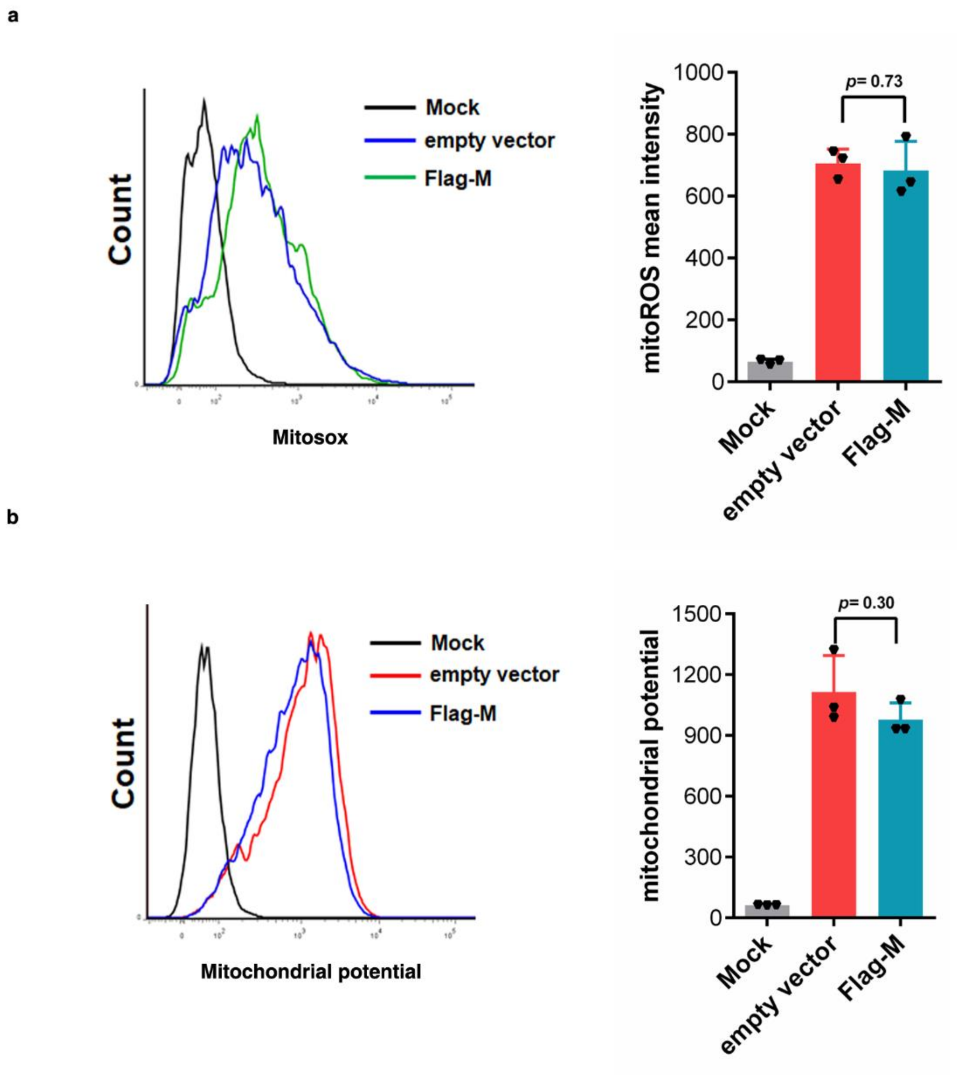

Fig. S6 Detection of superoxide in live cells using MitoSOX™ Red superoxide indicator and detection of mitochondrial membrane potential using MitoProbe TMRM. **a** Huh7.0 cells were transfected with Flag-M plasmid and cells were subsequently stained with MitoProbe TMRM and analyzed on an Attune NxT Flow Cytometer using a 561 nm laser for excitation and 585 nm/16BP filter for emission. **b** Huh7.0 cells were transfected with Flag-M plasmid and cells were subsequently stained with MitoSOX™ Red superoxide indicator and analyzed on an Attune NxT Flow Cytometer using a 561 nm laser for excitation and 585 nm/16BP filter for emission. Three independent experiments with three technical repetitions were performed. Data are expressed as mean ± SEM (error bars). Statistical analyses used Student's *t* test. $P < 0.05$ was considered statistically significant.
